# Characteristics and outcome indicators in a specialist inpatient intellectual disability unit: an independent sector experience

**DOI:** 10.1192/bjo.2021.816

**Published:** 2021-06-18

**Authors:** Mike Apio, Witold Skalbania, Muhammad Aamer Khan, Bolanle Lotsu

**Affiliations:** Eldertree Lodge, The Huntercombe, Ashley

## Abstract

**Aims:**

To elucidate critical elements for effective outcomes in patients with complex and challenging behaviours admitted to specialist inpatient ‘locked rehabilitation’ intellectual disability unit (LRU).

**Background:**

People with intellectual disability of varying severity with or without associated mental disorder are at risk of deterioration presenting with problem behaviours at critical times of transition. In the context of their pre-set neurocognitive deficits, protective factors during early development include a robust psychosocial ‘parenting’ environment that optimises their strengths through nurturing and embedding a positive mind-set. Such environment is critical for the development of resilience as against reliance on external factors with high likelihood of change. The effect of early exposure to prenatal and or postnatal childhood adversities is a common denominator. The experience of abuse; from deprivation and neglect to physical violence and indeed sexual trauma predisposes to further perturbation and kindling effect on risks for early and later onset affective disorders. Specialist ID services become critical to the resetting of a distorted premorbid neuronal circuitry. A biopsychosocial approach to recreating a stable base and environmental enrichment may offer opportunities for enhancing neurocognitive remediation and enhance prosocial skills. Indicators for better outcomes may offer scope for focused intervention. This review highlights extent patients progress (response to treatment and symptom remission), length of Stay and discharge pathway could be predicated on their engagement with offered structured therapeutic activities.

**Method:**

Using a mixed model approach, 12-months data regarding patient characteristics, elements from HoNOs LD, with patient's self-reported experience and utilization of therapy, progress of patients in the service were reviewed to elucidate factors that may predict improved outcomes..

**Result:**

Of 48 patients, 18 females and 30 males identified in the 12-months from January 2019, 7 females were discharged/transferred with one stepped up to LSU and another side-moved to a LRU. 6 have identified places and 5 require ongoing care. Of the males, 8 were discharged and 5 have identified placements. 16 inpatients with support completed questionnaires (10 males, 6 females). Majority identified structured therapeutic activities as helpful in their progress. Data for length of stay ranged from 12 to over 120 months with a mean of 31 months ignoring potential discharges.

**Conclusion:**

Findings suggest patients able to engage in structured therapeutic activities in conjunction with concordance to treatment are more likely to progress earlier in their care.

